# Effect of Rearing System and Season on Herbage Intake, Performance and Meat Nutritional Quality of Growing Rabbits

**DOI:** 10.3390/ani16203218

**Published:** 2026-10-13

**Authors:** Luigia Bosa, Alessandro Dal Bosco, Simona Mattioli, Marta Vispi, Lorenzo Nompleggio, Gloria Bernabucci, Riccardo Primi, Cesare Castellini

**Affiliations:** 1 Department of Agricultural, Food and Environmental Sciences, University of Perugia, 06121 Perugia, Italy; alessandro.dalbosco@unipg.it (A.D.B.); lorenzo.nompleggio@studenti.unipg.it (L.N.);; 2 Department of Agriculture and Forest Sciences, University of Tuscia, 01100 Viterbo, Italy; gloria.bernabucci@unitus.it (G.B.);

**Keywords:** fatty acid profile, outdoor rearing, oxidative stability, rabbit meat, seasonal effects

## Abstract

Consumer demand for sustainable and animal-friendly systems has driven interest in alternative livestock management; however, its impact on productive performance and meat quality requires further validation. This study monitored outdoor-reared rabbits during the spring and autumn seasons over a 48 d experimental period, comparing this alternative system to cage-reared rabbits slaughtered at a fixed age (80 d). Forage and energy intakes were evaluated alongside a post-slaughter analysis of muscle lipid content, fatty acid profiles, and tocopherol deposition. Meat oxidative stability was assessed by measuring thiobarbituric acid reactive substances (TBARS). Data were analyzed using mixed linear models and Principal Component Analysis (PCA). The results demonstrate that pasture-based rabbit systems offer a suitable alternative to conventional cages. Although pasture access significantly reduced growth performance, with average daily gain dropping by up to 7.24 g/day and digestible energy intake decreasing by approximately 6.20 MJ in autumn-reared rabbits, it enhanced meat quality by yielding leaner muscle profiles and higher levels of n-3 polyunsaturated fatty acids (PUFAs). Furthermore, although pasture-based systems are widely reported to increase physical activity—potentially elevating metabolic rate and oxidative load—the concurrent intake of natural antioxidants from fresh forage effectively maintained high oxidative stability in meat. Ultimately, these findings validate pasture-based systems as a viable approach to delivering high-quality meat within sustainable, welfare-friendly production frameworks.

## Simple Summary

European rabbit farming is declining due to falling meat consumption, pet status perception, and animal welfare concerns over wire cage systems (currently housing >90% of rabbits). However, rabbit meat is recognized as a sustainable, low-fat protein source, and healthy meat option. In addition, unlike pigs or poultry, rabbits do not compete for human grain crops, and outdoor grazing further enhances their meat’s nutritional profile. These aspects are driving interest in outdoor, cage-free alternative systems for rabbits. The present study compares indoor cage farming with outdoor pasture-based systems for rabbits, investigating how spring and autumn seasons impact growth performance, herbage intake, and meat quality.

## 1. Introduction

In recent years, the European rabbit production sector has experienced a marked decline, driven by a reduction in meat consumption and an increasing public concern regarding intensive farming practices, particularly the use of cages [1,2]. Despite this trend, more than 90% of rabbits produced in Europe are still reared in wire cages [3]. Changing sociocultural perceptions may further accelerate this decline, as rabbits are increasingly perceived by the public as companion animals [4,5]. In response, the rabbit production sector has directed its efforts toward alternative production systems, aligning with the societal changes and the evolving European policies that promote a transition toward cage-free farming [1,6].

Within this context, the alternative systems proposed by various authors and institutions [1] are not intended to replace conventional rabbit production. Rather, they aim to diversify the production model by offering alternative choices to consumers. Consistent with this perspective, previous studies [7,8] demonstrated that rabbits reared in outdoor systems with access to grass pens exhibit a broader behavioural repertoire.

As social herbivores, rabbits benefit from environments that provide sufficient space to interact (or to avoid interaction) and allow the expression of specific behaviours like grazing [9]. While direct indicators of animal welfare were not evaluated in the present study, previous behavioural research suggests that providing rabbits with grazing opportunities enhances their behavioural repertoire [7,8], which may in turn improve public perception of rabbit farming.

From a broader production perspective, rabbit meat is recognized in the literature as an efficient protein source with a lower environmental footprint compared to other livestock [10,11,12]. As herbivores, rabbits do not directly compete with humans for food resources, unlike other monogastric species such as pigs and poultry [3,13], which rely on cereal- and soybean-based diets. In addition, rabbit meat is characterized by a low overall fat content and features a favourable lipid profile with a high proportion of polyunsaturated fatty acids (PUFAs), particularly of the n-3 series. These fatty acids, especially the long-chain PUFAs, are associated with lower risks of cardiovascular diseases [14,15]. Pasture-based production systems can further enhance the n-3 fatty acid content of rabbit meat, thereby improving its nutritional value, as fresh forage intake increases the proportion of n-3 PUFAs in the rabbit meat [16]. In organic rabbit farming, forage represents the most cost-effective feed resource, yet it remains one of the least understood, particularly regarding intake dynamics and nutritional value. A deeper understanding of forage intake is therefore essential to optimize grazing management, including pasture allocation and dietary supplementation with locally produced mixed cereal–legume crops or commercial pelleted feeds [17,18,19]. In a preliminary study, Martin et al. [17] estimated that forage accounted for 43% of total daily dry matter intake in rabbits grazing on an organic farm. Although rabbits can efficiently utilize forage and consume a large proportion of the available biomass, they exhibit a selective feeding behaviour, actively foraging on young leaves and protein-rich species [13,20]. Feed intake regulation in rabbits is primarily regulated by dietary energy concentration, with voluntary intake adjusted to maintain energy balance when digestible energy exceeds approximately 9.0 to 9.5 MJ per kg of dry matter [21]. However, data on forage intake in pasture-based systems remain limited, particularly regarding the influence of factors like grass availability and quality. Under their specific experimental conditions, Martin [17] and Plagnet [19] identified forage availability as the limiting factor conditioning grass intake. Nevertheless, a comprehensive understanding of how the production system and seasonal variations affect forage intake, growth performance, and meat quality in growing rabbits is still lacking. To further understand the feasibility of these pasture-based systems, the present experiment focuses specifically on quantifying herbage intake, growth performance, and meat quality under seasonal conditions.

Therefore, this study aimed to investigate the effects of two rearing systems (indoor cage vs. outdoor pasture-based) on the growth performance and meat nutritional characteristics of growing rabbits. Within the outdoor pasture-based system, herbage intake and seasonal variations (spring vs. autumn) were also evaluated.

## 2. Materials and Methods

### 2.1. Ethical Approval

All methods were reported in accordance with the ARRIVE guidelines [22] for the reporting of animal experiments. The experimental design complied with Italian legislation on animal welfare for experimental and scientific purposes [23] and was approved by the Ethics Committee of the University of Perugia (Protocol No. 349569, 17 October 2024).

At the end of the experimental period, rabbits were transported to an authorized slaughterhouse in accordance with Council Regulation (EC) No. 1099/2009 and relevant national legislation on the protection of animals at the time of slaughter. Animals were stunned prior to slaughter following standard commercial procedures.

### 2.2. Experimental Design, Animals and Rearing Condition

This study compared two rabbit production systems over two seasons (spring and autumn 2025):

- Indoor caged system;
- Outdoor pasture-based system.

A total of 160 New Zealand White (NZW) rabbits with a balanced sex ratio (1:1) were used. All animals were weaned at 30 days of age (mean initial body weight: 614.4 ± 8.2 g) and randomly allocated to the experimental groups.

The indoor caged group (n = 40 rabbits per season) was housed under controlled environmental conditions in a commercial rabbitry (Bagnoregio, Italy; 42.627° N, 12.088° E), located approximately 10 km from the outdoor experimental site. The animals were kept in monocellular cages (28 × 40 × 30 cm width × length × height) inside a closed building maintained at a constant temperature of 18 °C, humidity of 65–70% and a ventilation speed of 0.2–0.3 m/s. Feed and water were provided ad libitum.

The outdoor groups (n = 40 rabbits per season) were reared at the “Nello Lupori” experimental farm of the University of Tuscia (Viterbo, Italy; 42°25′36.1″ N, 12°4′49.3″ E) and allocated to 4 mobile shelters (10 rabbits per shelter per season) connected to an outdoor paddock via an access platform. Each shelter measured 2.0 × 1.0 × 0.70 m (length × width × height) and comprised two cage-style pens. The shelters featured a hot-dip galvanized steel frame with rear wheels for mobility, galvanized wire mesh sides, a PVC slatted floor, a feeder, a hay rack, and a removable polypropylene nest. An insulated roof provided protection from rain and sun, and a modular design allowed the pens to be joined at the rear. The prevalent environmental condition in the rearing zone was a transitional Mediterranean climate characterized by mild winters, warm-to-hot summers. In the spring, the average temperature was 18.9 °C and 65% RH, whereas in the autumn it was 16.7 °C and 68.3% RH.

**Figure 1:**
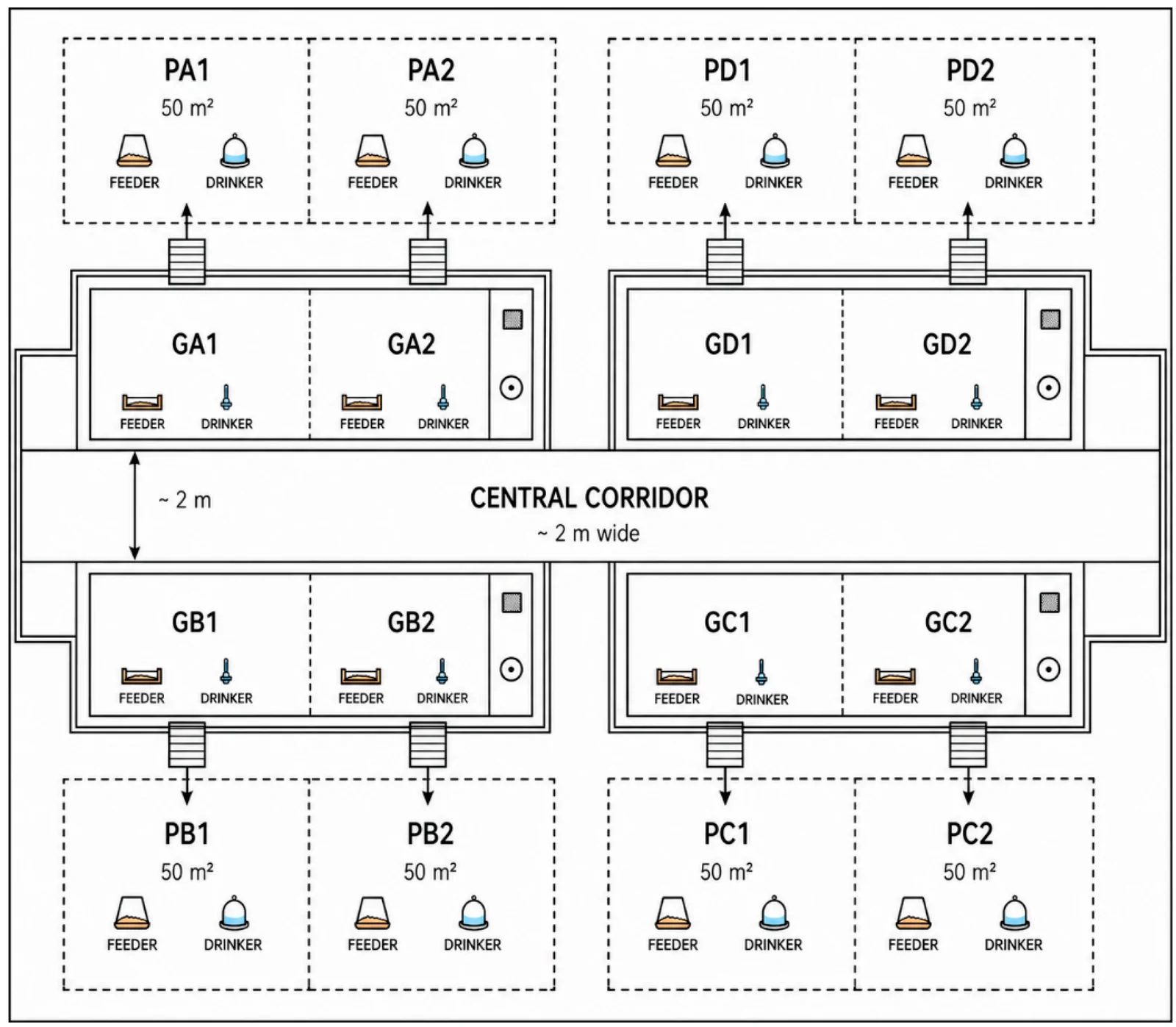
Schematic representation of the experimental housing system. Diagram of outdoor pens.

From the first 24 h after the transfer, the animals remained confined within the shelters with the access platforms closed to facilitate adaptation, and subsequently they were granted free access to the pastures. Each unit was equipped with a plastic feeder featuring anti-rodent protection and a stainless-steel nipple drinker connected to a water tank with a float valve (Figure 1). Although no paddock rotation was applied, paddocks remained vacant between seasons to allow adequate pasture recovery and mitigate potential sanitary risks. The experimental design complied with European organic production standards (Article 42 of Regulation EC No. 834/2007), which establish a minimum outdoor space of 5 m^2^ per growing rabbit.

The pasture was a natural permanent grassland characterized by a botanical composition typical of Central Italian meadows, predominantly composed of species belonging to the *Poaceae*, *Fabaceae*, and *Asteraceae* families, alongside a minor proportion of other local forbs. Each outdoor paddock measured 50 m^2^ (providing 5 m^2^ per rabbit), giving the animals unrestricted, 24 h access to the paddocks.

In both systems, rabbits always had ad libitum access to water and a commercial pelleted diet.

Schematic representation of the outdoor housing system. Each housing unit was connected to a 50 m^2^ outdoor paddock through an access ramp. Units GA1, GB1, GC1, and GD1, together with their corresponding paddocks, were used to house 10 rabbits each in different seasons. Each unit and paddock was equipped with a feeder and a drinker. A central corridor approximately 2 m wide allowed routine management operations.

### 2.3. Feeding, Growth, and Health Measurements

The commercial diet was formulated to meet the nutritional requirements of growing rabbits [24]. The same pelleted feed was provided to all experimental groups, but the indoor caged rabbits were fed exclusively on the pellets, whereas outdoor rabbits had access to both the pelleted diet and pasture. The pelleted diet intake was measured daily by weighing the offered feed and its refusals. Representative samples of the pelleted feed and pasture biomass were collected weekly for subsequent chemical analysis.

Individual body weight was recorded weekly. Growth was evaluated between 32 and 80 days of age, and the feed conversion ratio (FCR) was calculated as the ratio between total dry matter intake and body weight gain during this period. Animal health status and mortality were monitored daily.

### 2.4. Forage Intake Estimation

Forage intake was estimated using a modification of the method described by Lantinga [25], employing exclusion pens (0.50 × 0.50 m) to monitor grazed versus undisturbed herbage mass over a 48-day grazing period. To account for dynamic, non-linear pasture regrowth during the trial, biomass measurement was estimated according to (1)

(1) $$\mathrm{Grass}\;\mathrm{Intake}=(\mathrm{GMs}-\mathrm{GMe})+(\frac{\frac{1-\mathrm{GMe}}{\mathrm{GMs}}}{-\mathrm{In}(\frac{\mathrm{GMe}}{\mathrm{GMs}})})\times (\mathrm{GMu}-\mathrm{GMs})$$

where GMs is the herbage mass before rabbit grazing, GMe is the residual herbage mass at the end of the trial, and GMu is the undisturbed herbage mass measured in the exclusion pens.

### 2.5. Slaughtering and Meat Sampling

At 80 days of age, rabbits (n adjusted for mortality) were slaughtered in an EU-approved slaughterhouse after 12 h of feed withdrawal. Slaughtering was performed by exsanguination (carotid and jugular cutting) following electrical stunning. Twenty-four carcasses per experimental group were processed according to the recommendations of Blasco and Ouhayoun [26]. The recorded parameters included the carcass weight, carcass yield, interscapular and perivisceral fat.

After 24 h at 4 °C, the Longissimus thoracis et lumborum (LTL) muscles (between the 1st and 7th lumbar vertebrae) were excised from both sides of each carcass. One sample was used for physical analysis while the other was stored at −80 °C, awaiting chemical analyses.

### 2.6. Chemical and Proximate Analyses

The chemical composition of the commercial pelleted diet, pasture, and meat samples was determined according to standard AOAC [27] procedures. Feed and pasture samples were analyzed for dry matter (DM; method 934.01), crude protein (CP; method 984.13), ether extract (EE; method 920.39), ash (method 942.05), and crude fibre (CF; method 978.10). Structural fibre fractions, including Neutral Detergent Fibre (NDF), Acid Detergent Fibre (ADF), and Acid Detergent Lignin (ADL), were determined according to Van Soest et al. [28]. The digestible energy (DE, MJ/kg DM) of the pelleted diet and spring and autumn pasture was estimated using the (2) as described by Fernández-Carmona et al. [29]:

DE = 14.2 − 0.205 × ADF + 0.218 × EE + 0.057 × CP(2)

where ADF, EE, and CP are expressed as a percentage of DM.

Daily DE intake from both the pelleted diet and the pasture was calculated by multiplying the daily DM intake (kg DM/day) by the respective estimated DE concentration (MJ/kg DM) of each feed source. Total daily DE intake was subsequently obtained as the sum of the DE intakes from the pelleted diet and pasture.

The proximate composition of the LTL muscle was determined on duplicate samples following official AOAC [27] protocols. Briefly, moisture content was measured by oven-drying the meat samples at 125 °C for 24 h (method 950.46).

The total lipid content of meat samples was determined gravimetrically via ether extraction using a Soxhlet apparatus (method 960.30). Crude protein was analyzed using the Kjeldahl method (method 992.15), and ash content was measured by incinerating samples in a muffle furnace at 600 °C until a constant weight was achieved (method 923.03).

### 2.7. Analysis of Fatty Acid Methyl Esters

Fatty acid methyl esters (FAME) were prepared from the commercial pelleted diet, pasture (spring and autumn) and the LTL muscle samples, and analyzed by gas chromatography (Varian CP-3800, equipped with a flame ionization detector FID). Fatty acids were identified by comparison with commercial standards (Supelco37 Component FAME Mix, Sigma-Aldrich, St. Louis, MO, USA) according to previously described procedures.

Results were expressed as a percentage for the pelleted diet and pasture and in mg/100 g for meat samples. Fatty acid content, including saturated fatty acids (SFA), monounsaturated fatty acids (MUFAs), n-3 and n-6 PUFAs, and long-chain PUFAs (LCPs; over 20 carbons) and the n-3/n-6 ratio were calculated based on the fatty acid profiles.

Additionally, the daily intake of linoleic acid (LA, C18:2 n-6) and α-linolenic acid (ALA, C18:3 n-3) was estimated by integrating the FAME composition data of the pelleted diet and pasture with their respective consumption levels.

### 2.8. Vitamins and Lipid Oxidation of Meat

Tocopherols and tocotrienols were quantified using HPLC according to Hewavitharana et al. [30]. Samples were analyzed using an HPLC system equipped with a fluorescence detector and a Synergi Hydro-RP column (4 μm, 4.6 × 100 mm; Phenomenex, Bologna, Italy). The injection volume was 50 μL, the flow rate was 2.0 mL/min, and fluorescence detection was performed at excitation and emission wavelengths of 295 and 328 nm, respectively. Lipid oxidation in meat was assessed using the thiobarbituric acid reactive substances method [31] and expressed as µg of malondialdehyde per g of meat. Absorbance was measured at 532 nm using a spectrophotometer (Hitachi U-2000, Hitachi Ltd., Tokyo, Japan).

### 2.9. Nutritional Indexes

The Atherogenic Index (AI) and Thrombogenic Index (TI) were calculated according to Ulbricht and Southgate (1991) using the fatty acid composition of the meat, while the Healthy Fatty Index (HFI) was calculated according to Dal Bosco et al. [32]. The Δ5- and Δ6-desaturase activities were estimated according to methods previously described by [32] from the corresponding product-to-precursor fatty acid ratios.

### 2.10. Statistical Analysis

Data were analyzed using a mixed linear model in STATA 19 [33], tailored to the experimental scale of the different variables. For individual-level variables (e.g., live weight, carcass traits), the fixed effects included the rearing system (indoor vs. outdoor pasture), season (S: spring vs. autumn), and their interaction. To account for the hierarchical structure of the data and prevent pseudoreplication, the housing unit (individual cage for the indoor system, or pen for the outdoor system) was included as a random effect, within which individual animals were nested. For variables measured at the group level within the outdoor system (i.e., pasture intake and feed consumption), the pen was defined as the experimental unit. These variables were analyzed using a restricted model evaluating the fixed effect of Season.

Least squares means were compared using the Bonferroni adjustment, with statistical significance set at *p* < 0.05. For all models, underlying assumptions of residual normality and homoscedasticity were verified through visual inspection (quantile-quantile and studentized residual vs. fitted value plots) and using a Breusch–Pagan/Cook–Weisberg test.

The effect of sex was evaluated preliminarily but was excluded from the final model because it was not significant.

Mortality was analyzed using a chi-square test of independence to evaluate differences among groups (system effect: indoor vs. outdoor in spring; season effect: spring vs. autumn in the outdoor system). Because expected frequencies were low, Fisher’s exact test was applied. Statistical significance was set at *p* < 0.05.

Principal Component Analysis (PCA) was performed to evaluate the relationships between the daily dietary intake of the PUFA precursors (LA and ALA, respectively) and the concentrations of n-3 and n-6 LCP, tocopherols, and thiobarbituric acid reactive substances (TBARS) in the LTL muscle. PCA was executed using the PCA procedure in STATA 19 [33].

## 3. Results and Discussion

### 3.1. Chemical Composition of the Pelleted Diet and Pasture

The chemical composition of the pelleted diet and pasture showed marked differences in the main components (Table 1). Pasture was characterized by a lower DM content and, consequently, lower concentrations of CP, EE, and ash per kg. Fibre content and fibre fractions also differed substantially, resulting in a markedly lower DE compared to the pelleted diet (11.26 ± 0.46 vs. 9.06 ± 0.35 and 7.51 ± 0.30 MJ/kg of DM for the pelleted diet, pasture spring and pasture autumn, respectively; *p* < 0.003). Almost the same trend was observed for the tocopherol content. Within the pasture, estimated DE values varied between seasons, with higher values observed in spring than in autumn (9.06 vs. 7.51 MJ/kg DM, respectively). These seasonal differences are consistent with seasonal changes in plant maturity and composition, which influence forage nutritive value [18].

The fatty acid composition also differed significantly between pelleted diet and pasture, particularly in the ratio of essential fatty acids. In the pelleted diet, linoleic acid was predominant, whereas pasture was characterized by a higher proportion of α-linolenic acid, in agreement with previous studies [8,34]. Although pasture plants contain very little total fat, the fat they do have is exceptionally high quality, consisting mostly of n-3 polyunsaturated fatty acids. In leaf tissues, galactolipids are highly unsaturated and predominantly composed of ALA (up to 87–94%), while sulfolipids contain approximately equal proportions of palmitic and linolenic acids [35]. The fatty acid profile of pasture is generally characterized by a high proportion of α-linolenic acid (ALA), although its composition varies according to botanical composition, phenological stage, and environmental conditions. In the present study, the ALA content in the pasture is about 10-fold higher (11.56 in spring and 12.00 in autumn) than that contained in the pelleted feed (1.95% ± 1.24) when expressed on a fresh matter basis (i.e., the actual intake of rabbits).

Seasonal variation further influences forage fatty acid composition, with higher ALA concentrations typically observed in spring [36]. However, information on fatty acid variability in forage species, maturity stages, and conservation methods remains limited [37].

These differences were reflected in the diets used in the present study, where the n-6/n-3 ratio was markedly higher in the pasture during spring compared to autumn (0.24 and 0.46 respectively).

### 3.2. Productive Traits

Both the production system and season significantly affected growth of rabbits (Table 2), whereas mortality was not influenced by either factor (χ^2^ = 0, df = 2, *p* = 1.00).

Cage-reared rabbits showed higher final body weight and ADG compared to pasture-reared rabbits (41.55–41.62 vs. 34.38–40.22 g/day). In line with previous literature [10,38,39], this difference could be hypothetically attributed to lower physical activity and energy expenditure in confined cages, allowing a greater proportion of dietary energy to be retained as fat deposition [10,38,39].

These differences can be explained through energy balance mechanisms. Body weight gain depends on the relationship between energy intake, energy expenditure (maintenance, activity, thermoregulation), and energy retention in body tissues. In cage-reared rabbits, lower energy expenditure associated with physical activity allows a greater proportion of dietary energy to be retained as scapular and perirenal fat deposition (1.58 and 1.55 vs. 1.18 and 0.85% of carcass weight, respectively; Table 2) and lipid (1.76 and 1.75 vs. 0.72 and 0.64 g/100 g muscle in spring and autumn, respectively; Table 3) observed in caged-reared rabbits.

Seasonal effects were evident in outdoor rabbits, with spring-reared individuals exhibiting higher growth rates than those reared in autumn. Based on energy balance estimates, the autumn-reared rabbits experienced a reduced energy intake of approximately 6.25 MJ over 48 days. This difference is driven by higher pasture intake during the spring, which positively affects growth rates. This finding is consistent with prior observations identifying herbage availability and composition as key determinants of forage intake [13,17]. Furthermore, increased pasture intake also increased protein intake, thereby improving ADG and final body weight compared to the autumn season, a trend supported by other studies.

Overall, the results suggest that cage systems favour body weight gain, whereas pasture alters feed intake and energy utilization, highlighting a trade-off between productive performance and kinetic activity characteristics of more extensive production systems.

### 3.3. Proximate Composition and Fatty Acid Profile of Meat

The proximate composition of the LTL muscle was marginally affected by rearing system (Table 3), except for lipid content. Pasture-reared rabbits had lower muscle fat, likely due to lower slaughter weight, reduced pelleted feed intake, and increased activity [16,40].

These results are consistent with previous studies showing that the inclusion of fresh or dehydrated alfalfa in rabbit diets reduces lipid deposition [16,39,40].

The fatty acid (FA) profile of LTL muscle was significantly influenced by rearing system and season (Table 4). Since FA concentrations are expressed as mg/100 g of muscle, they were also influenced by the intramuscular fat content. Control rabbits in both seasons showed higher concentrations of total saturated fatty acids (SFAs; 579.9–581.9 vs. 213.8–228.1 mg/100 g muscle; *p* < 0.001), and linoleic acid (385.5–386.9 vs. 104.4–125.1 mg/100 g muscle; *p* < 0.001). In contrast, pasture-reared rabbits, particularly in spring, had higher concentrations of α-linolenic acid (40.3 vs. 18.0 mg/100 g muscle; *p* < 0.001), eicosapentaenoic acid (13.5 vs. 1.0 mg/100 g muscle; *p* < 0.001), docosahexaenoic acid (16.8–19.2 vs. 0.7 mg/100 g muscle; *p* < 0.001), and total n-3 PUFAs (93.6 vs. 38.6 mg/100 g muscle; *p* < 0.001).

These findings confirm the positive effect of pasture intake on the enriching of rabbit meat with n-3 fatty acids. Similar results were reported in pasture-reared rabbits by Forrester-Anderson et al. [41], who observed higher levels of long-chain n-3 fatty acids, such as EPA and DHA and lower fat content. Comparable improvements in fatty acid profiles have also been reported in grass-fed cattle [42,43] and in pasture-reared rabbits [39]. Consequently, the n-6/n-3 ratio was substantially lower in pasture-reared rabbits (1.83–1.85) than in caged rabbits (11.84–11.88; Table 5), indicating an improved nutritional quality of the lipid fraction.

Other nutritional indexes further highlighted these differences. Accordingly, the atherogenic (AI) and thrombogenic (TI) indexes, which widely indicate the potential impact of dietary lipids on cardiovascular health, were lower in pasture-reared rabbits than in the control group. Furthermore, a novel nutritional index specifically designed for meat products, the healthy fatty acid index (HFI), was higher in pasture-reared rabbits, supporting the overall improvement in meat lipid quality. The estimated ∆5- and ∆6-desaturase activities were higher in pasture-reared rabbits, suggesting an enhanced endogenous synthesis of long-chain polyunsaturated (LCP) n-3 fatty acids, given that the Δ6-desaturase is considered the rate-limiting enzyme of this metabolic pathway [44]. The biosynthesis of docosahexaenoic acid (DHA) involves the elongation of C22:5n-3 to C24:5n-3, followed by Δ6-desaturation and peroxisomal β-oxidation [45]. This aligns with the well-known competition between n-3 and n-6 fatty acids for desaturase enzymes [44,45,46].

### 3.4. Antioxidant Status of Meat

Pasture intake significantly influenced the antioxidant content of the meat (Table 6). Total tocopherol levels, specifically α-tocopherol, were higher in pasture-reared rabbits, whereas TBARS values remained unaffected. Although higher muscle α-tocopherol concentrations are generally associated with enhanced oxidative stability [47], this relationship is not always proportional, as lipid oxidation is also driven by fatty acid unsaturation and other pro- and antioxidant factors [48]. Mattioli et al. [49] demonstrated that in outdoor-reared chickens, higher foraging intake (primarily of tocopherols and carotenoids) is often accompanied by increased kinetic activity, which triggers an “oxidative thrust”. While the resulting overproduction of free radicals can challenge in vivo antioxidant defences, it is typically neutralized by the high antioxidant intake from foraging. This balance prevents oxidative damage in the meat, particularly in highly active genotypes. Notably, fresh grass contains four to six times more α-tocopherol and two to eight times more carotenoids than standard pelleted feed [50]. Therefore, the stable TBARS levels observed in this study may be attributed to this balancing mechanism, supported by the 1.5-fold higher tocopherol content found in rabbits with pasture access.

### 3.5. Principal Component Analysis (PCA)

**Figure 2:**
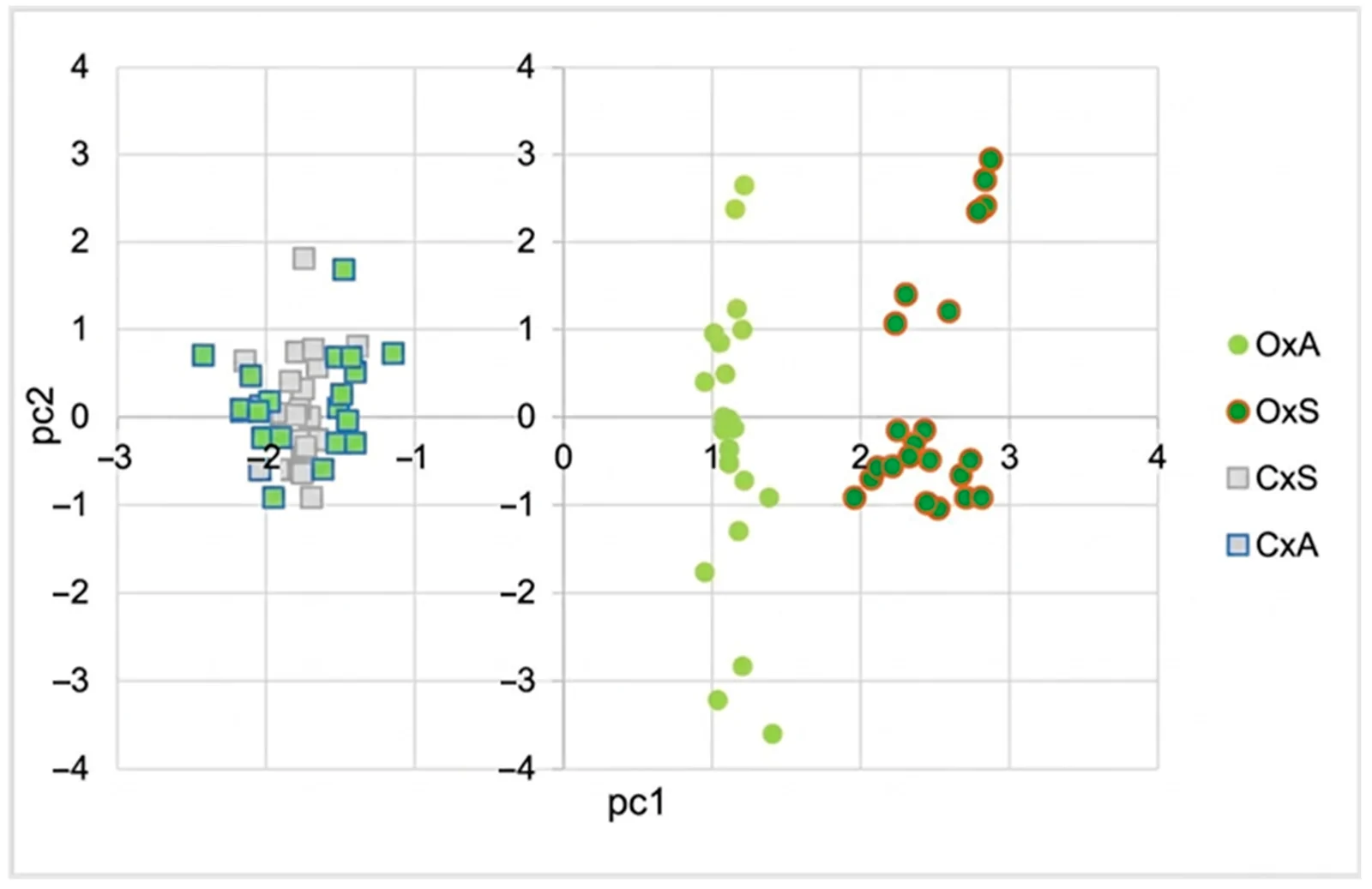
Principal Component Analysis between the total dietary intake of linoleic acid and α-linolenic acid and the n-3 and n-6 LCP in LTL muscle. Principal Component 1 (pc1) explains 53.48% of the total variance, Principal Component 2 (pc2) explains 22.85% of the variance. Legend: O = outdoor; C = cage; A = autumn, S = spring.

The PCA results (Figure 2) highlighted the association between the total dietary intake of linoleic acid and α-linolenic acid and the n-3 and n-6 LCP in LTL muscle. Principal Component Analysis revealed that Component 1 (53.48% of the total variance explained) contrasted a higher dietary intake of ALA with higher concentrations of long-chain n-3 in meat, whereas dietary LA intake was associated with long-chain n-6 PUFAs, thereby discriminating both the rearing systems and the seasonal effect. These patterns confirm that pasture intake effectively promotes the enrichment of rabbit meat with n-3 fatty acids.

The second principal component (PC2, explaining 22.85% of the variance) demonstrated an inverse relationship between tocopherols and TBARS. This indicates that the dietary intake of natural antioxidants effectively helped to maintain the oxidative stability of rabbit meat, despite its elevated levels of LCP.

Although locomotor activity was not directly measured in the present study, previous research clearly indicates that free-range rabbits display enhanced physical activity and spatial movement compared to caged counterparts [7,8]. We hypothesize that a similar increase in physical activity in outdoor rabbits may have contributed to higher lipid β-oxidation and muscular oxygen turnover, although this potential mechanism warrants confirmation through direct behavioural tracking in future trials.

Similarly, in our study, the expected higher oxidative pressure in outdoor animals was successfully countered by the accumulation of antioxidants in the muscle tissue (e.g., tocopherols), which preserved the structural integrity of long-chain n-3 fatty acids, as indicated by the high PC1 loading. This physiological balance ensures that, despite the well-known susceptibility of polyunsaturated fats to oxidation [40,51], the meat successfully maintains both its high nutritional quality and its oxidative stability, as reflected by the low TBARS values.

## 4. Conclusions

Although pasture-based rearing resulted in a lower final body weight at the fixed slaughter age, thereby reducing overall growth performance, it significantly enhanced the nutritional quality of rabbit meat.

The key findings demonstrate a reduced muscle lipid content—resulting in a leaner meat product—alongside a substantial enrichment in long-chain n-3 PUFAs, driven by the high dietary intake of pasture. Furthermore, the elevated accumulation of α-tocopherol provided effective antioxidant protection, mitigating lipid oxidation and ensuring the oxidative stability of the meat despite its higher degree of unsaturation.

Overall, these results highlight pasture-based systems as an effective strategy to produce high-quality rabbit meat characterized by an optimized fatty acid profile and improved antioxidant status. While these findings suggest that outdoor pasture access offers potential benefits for alternative farming systems, further research, incorporating direct animal-based welfare indicators and life-cycle environmental assessments, is warranted to fully evaluate the overall welfare and sustainability of outdoor rabbit production across different seasons and pasture types.

## Figures and Tables

**Table 1:** Chemical composition of pelleted diets and pastures (% DM; Least-Squares Means ± Standard Deviations).

Parameter	Pelleted Diet	Pasture Spring	Pasture Autumn	*p*-Value
n	3	4	4	
Dry matter (%)	88.1 ± 2.01 a	20.0 ± 1.72 c	27.8 ± 1.80 b	<0.001
Crude protein (% DM)	18.7 ± 1.10 b	21.8 ± 1.00 a	10.2 ± 1.05 c	<0.001
Crude fibre (% DM)	17.8 ± 1.22 a	3.5 ± 0.62 c	4.5 ± 0.60 b	<0.001
Ether extract (% DM)	3.57 ± 0.19 a	1.80 ± 0.18 c	2.50 ± 0.17 b	0.01
Ash (% DM)	7.38 ± 0.39 a	8.16 ± 0.24 b	9.18 ± 0.20 b	<0.001
NDF (% DM)	28.8 ± 1.18 c	47.0 ± 2.00 b	58.0 ± 2.25 a	<0.001
ADF (%)	18.5 ± 1.45 c	27.0 ± 1.24 b	35.0 ± 1.50 a	0.003
Hemicellulose (% DM)	10.0 ± 0.95 b	20.0 ± 1.10 b	23.0 ± 1.12 a	0.004
Cellulose (% DM)	14.0 ± 1.00 c	20.8 ± 1.08 b	26.0 ± 1.25 a	0.005
ADL (% DM)	4.10 ± 0.42 b	5.50 ± 0.28 b	8.11 ± a 0.30	<0.001
DE (MJ/kg DM)	11.26 ± 0.46 a	9.06 ± 0.35 b	7.51 ± 0.30 c	0.003
Tocopherols (mg/kg DM)	41 ± 2 a	154 ± 8 b	62 ± 3 a	0.005
Fatty acid profile (%)				
C16:0 (Palmitic)	15.02 ± 0.65 b	13.55 ± 0.60 b	18.42 ± 0.71 a	0.005
C18:0 (Stearic)	3.81 ± 0.20 a	0.94 ± 0.11 c	1.54 ± 0.15 b	<0.001
C18:1n-9 (Oleic)	21.00 ± 1.35 a	0.95 ± 0.94 b	1.32 ± 0.85 b	<0.001
C18:2n-6 (LA)	40.58 ± 2.15 a	13.95 ± 1.85 c	19.98 ± 1.87 b	<0.001
C18:3n-3 (ALA)	1.95 ± 1.24 c	57.80 ± 2.84 a	43.20 ± 2.48 b	<0.001
n-6/n-3 ratio	20.81 ± 2.24 a	0.24 ± 0.15 c	0.46 ± 0.24 b	<0.001

DM = dry matter; DE = digestible energy. Means in the same row with different letters (a–c) differ significantly (*p* < 0.05). DE was estimated according to Fernandez-Carmona et al. [29], using the following equation: DE (MJ/kg DM) = 14.2 − 0.205 × ADF + 0.218 × EE + 0.057 × CP.

**Table 2:** Productive performance and estimated digestible energy (DE) intake of rabbits under different rearing systems and seasons.

	Control		Pasture		*p*-Value		
Parameter	Spring	Autumn	Spring	Autumn	Rearing System (RS)	Season (S)	RxS
Initial weight (g)	605 ± 15.1	610 ± 13.0	620 ± 12.7	619 ± 10.1	0.623	0.510	0.420
Final weight (80 d) (g)	2570 ± 50.2 b	2560 ± 24.8 b	2457 ± 45.6 ab	2360 ± 7.5 a	0.019	0.996	0.233
ADG from 32 to 80 d (g/d)	40.68 ± 1.09 b	40.60 ± 0.42 b	38.30 ± 0.82 ab	36.25 ± 0.13 a	0.003	0.986	0.239
Carcass weight (g)	1479 ± 15.3 c	1477 ± 18.2 c	1394 ± 17.3 b	1331 ± 15.8 a	<0.001	0.990	0.015
Dressing percentage (%)	57.54 ± 0.85 b	57.76 ± 0.83 b	56.72 ± 0.84 a	56.60 ± 0.80 a	0.012	0.350	0.063
Scapular & perirenal fat (% carcass)	1.58 ± 0.04 b	1.55 ± 0.04 b	1.18 ± 0.03 b	0.85 ± 0.03 a	<0.001	0.045	0.005
Mortality rate (%, n)	5.0 (2/40)	2.5 (1/40)	5.0 (2/40)	5.0 (2/40)	1.000	1.000	—
Feed Index	2.90 ± 0.08 b	2.93 ± 0.09 b	2.42 ± 0.07 a	2.68 ± 0.07 ab	<0.005	0.083	<0.001
Feed intake (g DM/d)	119.1 ± 1.15 c	120.0 ± 1.10 c	92.5 ± 0.87 a	97.3 ± 0.92 b	<0.001	0.980	<0.001
Pasture intake (g DM/d)	—	—	21.10 ± 0.37 b	15.37 ± 0.37 a	N.A.	<0.001	N.A.
Total intake (g DM/d)	119.1 ± 1.15 b	120.0 ± 1.10 b	113.5 ± 0.87 a	112.6 ± 0.90 a	<0.001	0.248	<0.001
DE intake grass (MJ/d)	—	—	0.20 ± 0.01 b	0.12 ± 0.01 a	N.A.	<0.001	N.A.
Total DE intake (MJ/d)	1.34 ± 0.01 b	1.35 ± 0.01 b	1.24 ± 0.01 a	1.21 ± 0.01 a	0.001	0.159	0.001

N = 40/group; N.A. = not applicable; DM = dry matter; values are expressed as mean ± SE. —: data not determined. Different letters (a–c) within a row indicate significant differences (*p* < 0.05) according to Bonferroni-adjusted pairwise comparisons. For individual-level traits (weights, ADG, fat deposit), the independent experimental units are cages for indoor (*N* = 40 per season) and animals nested within pens for outdoor (*N* = 40 animals clustered across four pens per season). For pasture and intake-exclusive metrics, only the outdoor system was analyzed to test the seasonal effect (*N* = 4 independent pens per season). *p*-values refer to the overall effect tested using a mixed linear model.

**Table 3:** Chemical composition of LTL muscle (g/100 g muscle), of rabbits under different rearing systems and seasons.

	Control		Pasture		*p*-Value		
Parameter	Spring	Autumn	Spring	Autumn	Rearing System (RS)	Season (S)	RxS
Moisture	74.17 ± 0.90	74.33 ± 0.71	75.05 ± 0.75	75.10 ± 0.60	0.310	0.254	0.241
Protein	22.32 ± 0.35	22.30 ± 0.33	22.65 ± 0.29	22.39 ± 0.23	0.101	0.347	0.588
Lipids	1.76 ± 0.03 c	1.75 ± 0.04 c	0.72 ± 0.03 b	0.64 ± 0.02 a	0.003	0.150	0.070
Ash	1.75 ± 0.08	1.77 ± 0.07	1.58 ± 0.07	1.87 ± 0.05	0.264	0.749	0.120

N = 24/per group; values are expressed as mean ± SE. Different letters (a–c) within a row indicate significant differences (*p* < 0.05) according to Bonferroni-adjusted pairwise comparisons. *p*-values refer to the overall effect tested using a mixed linear model.

**Table 4:** Fatty acid profile of LTL muscle (mg/100 g muscle), of rabbits under different rearing systems and seasons.

	Control		Pasture		*p*-Value		
	Spring	Autumn	Spring	Autumn	Rearing System (RS)	Season (S)	RxS
C14:0	26.22 ± 1.02 a	26.31 ± 0.85 a	6.10 ± 0.86 b	7.85 ± 0.69 b	<0.001	0.835	0.123
C16:0	418.47 ± 7.85 a	416.63 ± 5.36 a	143.38 ± 6.60 b	154.83 ± 5.26 b	<0.001	0.136	0.266
C16:1cis9	28.04 ± 2.01 a	28.82 ± 1.28 a	3.528 ± 1.75 b	6.60 ± 1.39 b	<0.001	0.229	0.243
C18:0	119.02 ± 3.84 a	120.29 ± 3.55 a	59.91 ± 4.15 b	57.25 ± 3.31 b	<0.001	0.672	0.532
C18:1n-9	328.65 ± 8.32 a	328.07 ± 9.10 a	143.17 ± 7.77 b	146.86 ± 6.20 b	<0.001	0.588	0.812
C18:2n-6 LA	386.89 ± 7.01 a	385.47 ± 5.84 a	125.14 ± 5.18 b	104.43 ± 4.28 c	<0.001	0.012	0.007
C18:3n-3 α-ALA	17.99 ± 1.99 c	18.04 ± 2.12 c	40.33 ± 2.16 a	32.96 ± 1.54 b	<0.001	0.004	0.004
C20:4n-6 AA	64.35 ± 3.41 a	62.70 ± 2.17 a	42.61 ± 1.87 b	28.08 ± 1.74 c	<0.001	<0.001	<0.001
C20:5n-3 EPA	1.00 ± 0.64 c	0.99 ± 0.45 c	13.47 ± 0.30 a	3.31 ± 0.28 b	<0.001	<0.001	<0.001
C22:5n-3 DPAn-3	19.11 ± 0.63b	18.91 ± 0.36 b	19.17 ± 0.33 b	15.48 ± 0.42 a	0.002	<0.001	<0.001
C22:5n-6 DPAn-6	6.70 ± 0.17 a	6.74 ± 0.12 a	0.16 ± 0.09 b	0.28 ± 0.10 b	<0.001	0.432	0.626
C22:6n-3 DHA	0.74 ± 1.02 b	0.74 ± 0.92 b	16.80 ± 1.06 a	19.18 ± 0.85 a	<0.001	0.115	0.116
SFAs	579.91 ± 8.70 a	581.89 ± 7.93 a	213.84 ± 7.24 b	228.08 ± 6.57 b	<0.001	0.164	0.322
MUFAs	358.02 ± 9.74 a	360.54 ± 8.22 a	149.79 ± 8.01 b	160.54 ± 5.26 b	<0.001	0.241	0.385
PUFAs n-3	38.60 ± 2.44 b	38.71 ± 2.51 b	93.59 ± 2.48 a	72.27 ± 1.37 b	<0.001	<0.001	<0.001
PUFAs n-6	457.08 ± 5.85 a	458.12 ± 5.20 a	169.96 ± 6.40 b	132.80 ± 5.22 c	<0.001	<0.001	<0.001

N = 24/per group; values are expressed as mean ± SE. Different letters (a–c) within a row indicate significant differences (*p* < 0.05) according to Bonferroni-adjusted pairwise comparisons. *p*-values refer to the overall effect tested using a mixed linear model. LA: linoleic acid, ALA: α-linolenic acid, AA: arachidonic acid, EPA: eicosapentaenoic acid, DPA: docosapentaenoic acid, DHA: docosahexaenoic acid, SFAs: saturated fatty acids, MUFAs: monounsaturated fatty acids, PUFAs: polyunsaturated fatty acids.

**Table 5:** Fatty acid indexing of LTL meat of rabbits under different rearing systems and seasons.

Parameter	Control		Pasture		*p*-Value		
	Spring	Autumn	Spring	Autumn	Rearing System (RS)	Season (S)	RxS
n-6/n-3	11.88 ± 0.11 b	11.84 ± 0.10 b	1.83 ± 0.14 a	1.85 ± 0.11 a	0.001	0.909	0.905
Index of Atherogenicity	1.32 ± 0.02 b	1.33 ± 0.03 b	0.70 ± 0.02 a	0.84 ± 0.01 a	0.0089	<0.0001	0.0092
Index of Thrombogenicity	1.07 ± 0.02 b	1.08 ± 0.03 b	0.47 ± 0.02 a	0.61 ± 0.01 a	<0.0001	<0.0001	<0.0001
Healthy Fatty Index	3.21 ± 0.08 a	3.20 ± 0.10 a	5.05 ± 0.09 c	4.07 ± 0.10 b	<0.0001	<0.0001	<0.0001
∆5-desaturase ± ∆6-desaturase	0.15 ± 0.01 a	0.15 ± 0.01 a	0.31 ± 0.03 c	0.27 ± 0.01 b	0.0084	<0.0001	0.0072

N = 24/per group; values are expressed as mean ± SE. Different letters (a–c) within a row indicate significant differences (*p* < 0.05) according to Bonferroni-adjusted pairwise comparisons. *p*-values refer to the overall effect tested using a mixed linear model.

**Table 6:** Antioxidant status of LTL meat of rabbits under different rearing systems and seasons.

Parameter	Unit	Control		Pasture		*p*-Value		
		Spring	Autumn	Spring	Autumn	Rearing System (RS)	Season (S)	RxS
TBARS	μg MDA/g	0.08 ± 0.02	0.08 ± 0.02	0.10 ± 0.02	0.08 ± 0.01	0.163	0.224	0.211
δ-Tocopherol	μg/g	0.25 ± 0.05 b	0.26 ± 0.04 b	0.26 ± 0.05 b	0.11 ± 0.04 a	0.040	0.088	0.057
α-Tocopherol	μg/g	0.67 ± 0.29 b	0.66 ± 0.27 b	1.10 ± 0.20 a	1.20 ± 0.17 a	0.012	0.413	0.405
∑-Tocopherols	μg/g	0.92 ± 0.33 a	0.92 ± 0.33 a	1.36 ± 0.28 b	1.31 ± 0.22 b	0.022	0.961	0.946

N = 24 (per group); values are expressed as mean ± SE. Different letters (a, b) within a row indicate significant differences (*p* < 0.05) according to Bonferroni-adjusted pairwise comparisons. *p*-values refer to the overall effect tested using a mixed linear model.

## Data Availability

The data presented in this study are available on reasonable request from the corresponding author. The data are not publicly available due to institutional policy restrictions.
